# Combining Epidemiological and Genetic Networks Signifies the Importance of Early Treatment in HIV-1 Transmission

**DOI:** 10.1371/journal.pone.0046156

**Published:** 2012-09-28

**Authors:** Narges Zarrabi, Mattia Prosperi, Robert G. Belleman, Manuela Colafigli, Andrea De Luca, Peter M. A. Sloot

**Affiliations:** 1 Computational Science, University of Amsterdam, Amsterdam, The Netherlands; 2 College of Medicine, Department of Pathology, Immunology and Laboratory Medicine, Emerging Pathogens Institute, University of Florida, Gainesville, Florida, United States of America; 3 Clinic of Infectious Diseases, Catholic University of Sacred Heart, Rome, Italy; 4 Unit of Infectious Diseases, Siena University Hospital, Siena, Italy; 5 National Research University ITMO, St. Petersburg, Russia; 6 Nanyang Technological University, Singapore, Singapore; Centers for Disease Control and Prevention, United States of America

## Abstract

Inferring disease transmission networks is important in epidemiology in order to understand and prevent the spread of infectious diseases. Reconstruction of the infection transmission networks requires insight into viral genome data as well as social interactions. For the HIV-1 epidemic, current research either uses genetic information of patients' virus to infer the past infection events or uses statistics of sexual interactions to model the network structure of viral spreading. Methods for a reliable reconstruction of HIV-1 transmission dynamics, taking into account both molecular and societal data are still lacking. The aim of this study is to combine information from both genetic and epidemiological scales to characterize and analyse a transmission network of the HIV-1 epidemic in central Italy.

We introduce a novel filter-reduction method to build a network of HIV infected patients based on their social and treatment information. The network is then combined with a genetic network, to infer a hypothetical infection transmission network. We apply this method to a cohort study of HIV-1 infected patients in central Italy and find that patients who are highly connected in the network have longer untreated infection periods. We also find that the network structures for homosexual males and heterosexual populations are heterogeneous, consisting of a majority of ‘peripheral nodes’ that have only a few sexual interactions and a minority of ‘hub nodes’ that have many sexual interactions. Inferring HIV-1 transmission networks using this novel combined approach reveals remarkable correlations between high out-degree individuals and longer untreated infection periods. These findings signify the importance of early treatment and support the potential benefit of wide population screening, management of early diagnoses and anticipated antiretroviral treatment to prevent viral transmission and spread. The approach presented here for reconstructing HIV-1 transmission networks can have important repercussions in the design of intervention strategies for disease control.

## Introduction

Understanding the dynamics of infectious disease spreading demands a holistic approach [1]. Social interactions as well as genetic diversity of the transmitted viral agent among individuals dictate the dynamics of infectious disease spreading in a population. Hence, the infection transmission can be investigated at different spatio-temporal scales, from molecular to epidemiological levels.

At the epidemiological level, scientists have been trying to study the spread of infectious diseases using social or sexual contact networks, modelling the population as a complex network (where nodes are individuals and links are relationships) and running models of disease spread on top of that. In the case of type HIV-1 infection, these models have been used to understand the complexity of HIV-1 transmission and spread of viral drug resistance [2]–[8]. However, these models require estimation of many parameters such as frequency of sexual actions, transmission probability per action, and parameters that shape the network structure. For example, even though there is uncertainty about the network structures formed by social/sexual contacts, the network structure of ‘men who have sex with men’ (MSM) is assumed to be approximately scale-free with an exponent value in the range from 1.5 to 2.0 [6], [9]. Therefore, the degree distribution follows a power-law with a scaling factor equal to the exponent. A power-law distribution implies that low-degree nodes are many, whereas high-degree nodes are few [10], [11]. These assumptions however, are subject to change in different communities and cultures. Therefore the primary assumptions on the network structure and the choice of the uncertain parameter values to build a sexual contact network are still controversial.

Phylogenetic analysis has been employed to study the evolution of HIV-1 both at the population and intra-host level during different stages of the disease, using molecular sequences [12], [13]. Phylogenetic theory exploits genetic information of viruses and other species using mathematical methods of molecular evolution [14], [15]. Phylogenetic trees show the evolutionary relationships among genetic sequences in a population, where topology and branch lengths can be estimated via likelihood-based, parsimony-based or distance-based methods. Genetic isolates are placed at the leaves of these trees and the internal nodes are considered as hypothetical ancestors under a species' coalescence paradigm. Phylogenetic trees can be used to infer transmission clusters [16], [17], as well as temporal and spatial dynamics of the species' evolution, in a so-called *phylodynamic* framework [18], [19]. However, phylogenetic methods may not necessarily accurately represent the evolution of species and transmission of disease, both due to strong assumptions of the underlying mathematical models, and due to noise in the data. For instance, evolution of species is not always reducible to a tree form and a hierarchical tree may not represent the evolution of a species, such as in the case of recombination events [20]. Moreover, the agreement between phylogenetic reconstruction and epidemiological evidence of transmission events can be decreased due to other factors: in the case of HIV-1 infection, these include the long period of infectivity and convenient sampling (i.e. biased, non-uniform sampling in terms of locations or periods) [20], [21], [22].

This work proposes a new approach to combine information present at both genetic and epidemiological levels in order to obtain a more comprehensive picture of HIV-1 transmission. A filter-reduction method is applied to infer a meta-network of HIV-1 sequences based on the corresponding patient's demographic and medical information. For this meta-network, we use the term contact network as it contains all the contacts that are socially and sexually possible contact between infected individuals in the population. In contrast to standard network methods, no assumptions are being made on the network structure. An intersection of such contact network with a genetic distance network is subsequently computed, from which a hypothetical transmission network is inferred. The method is then applied to identify the HIV-1 subtype B transmission networks in central Italy. The structure of the inferred networks for the MSM and heterosexual risk groups is in agreement with the recognized network structures for social and sexual contacts in the HIV-1 infected population [23]. Moreover, highly connected patients in the network are found to be significantly correlated with longer periods without antiretroviral treatment.

Considering population level data beside genomic data is essential for understanding the true nature of infectious disease transmission networks, as was alluded to by DeGruttola et al. [24]. The approach presented here is, to the best of our knowledge, the first attempt to use both genetic and social information in order to characterise transmission networks for HIV-1.

## Results

### Characteristics of the study population

A dataset of 895 HIV-1 infected patients from a regional study cohort in Rome, Italy (see methods) was used in this study. Patients were divided into two separate groups according to their viral subtype: B and non-B subtype. One-hundred-twenty-two (13.5%) patients with a non-B subtype were excluded from the analysis. Of the 773 (86.5%) subtype B patients, 118 (15.3%) patients who had an unknown/other entry for the transmission group were also excluded from the analysis. Of the 655 patients included in the analysis, 65.0% were males and 35.0% females; HIV transmission risk categories were 27.0% MSM, 39.0% heterosexual contacts, 33.0% injecting drug users (IDU), 1.0% infected through blood products; 84.4% were Italian-born, 10.4% non-Italian born, while for 5.2% nation of birth was unknown. The median interquartile range (IQR) age was 48 (43–53) years; the median (IQR) calendar year of estimated seroconversion, an estimate of the start of the infection, was 1996 (1993–2000); the median (IQR) calendar year of viral genotyping was 2004 (2001–2007). At the time of viral genotyping, the overall median (IQR) plasma viral load was 4.1 log_10_ HIV RNA copies/ml (3.5–4.7). The percentage of therapy-naive patients was 19.3%, whilst 80.7% were antiretroviral therapy-experienced. The median (IQR) time from the estimated seroconversion date to the first viral sequence date was 8 (4–11) years. In the subset of therapy-experienced patients, the median (IQR) time from the estimated seroconversion date to the first therapy date was 3 (1.25–5) years, and the median (IQR) time passed from the first therapy date to the viral sequencing date was 4 (1–8) years.

### Filter-reduction method and network construction

**Figure 1:**
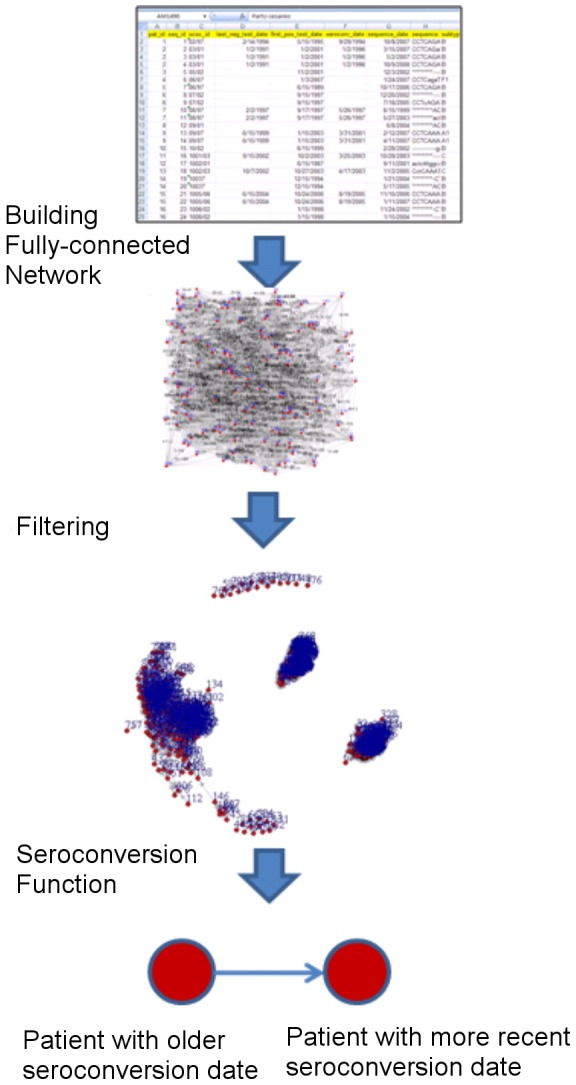
Workflow for constructing networks using the filter-reduction method. Starting from an undirected fully-connected network of all HIV sequences in the data, a set of social/sexual filters is applied to obtain an undirected filtered network. To convert the network to a directed one a seroconversion function is applied, deriving a contact network.

We proposed a new filter-reduction method to infer networks of HIV infected patients, taking into account patients attributes and parameters from literature. The filter-reduction method was defined as follows. Consider a social-sexual network as a graph/network composed of *N* nodes, *V(N)*. We started with an undirected fully-connected network of *V(N)* in which there is a link between each pair of nodes. A set of filters *F* was applied to the fully-connected network, reducing the number of edges through the filtering process. Depending on the data and type of the network the filtering process could vary. For building the network, we used HIV-1 sequence data that were annotated with demographical information and we applied a set of *social filters* (Table 1). The social filters were basic epidemiological criteria such as belonging to a similar age range (filter 1) and similar transmission risk group (filter 2), and the effect of treatment in reducing the transmission probability (filter 3). A direct connection between every two nodes that did not satisfy the epidemiological criteria was removed from the network. Table 1 summarizes the specific filtering rules used for reduction of the associated contact network (For details on the filtering process see Material and Methods). An undirected contact network is derived through the filtering process. For the heterosexual population a bipartite network is derived. This is an effect of rule b in of the second filter, in which we consider two populations with different genders, *males* (*g1*) and *females* (*g2*), and only links between different genders are allowed. A seroconversion function is applied to convert the undirected network to a directed one. The seroconversion function is based on patient's estimated seroconversion date and assigns the direction from a patient with an older seroconversion date to a patient with a more recent seroconversion date. The function results in having no directed cycles in the networks, meaning that there is no way to start at some vertex *v* and follow a sequence of edges that loops back to *v* again. Hence, the inferred network is a directed acyclic graph (DAG), a directed graph with no directed cycles [25]. DAGs are suitable to study and model processes in which information flows in a consistent direction through the network such as disease transmission [26], [27]. In the case of HIV-1, a “super-infection” may rarely occur, in which a patient is infected twice with two different virus strains (from different donors). However, it is highly unlikely that a patient is infected back with a variation of its own virus. In DAG, it is possible that nodes receive more than one incoming-link (the case of super-infection) but, since there are no directed cycles in the network, a node would never be re-infected with a variation of its own virus. Figure 1 shows the workflow for constructing networks using the filter reduction method.

**Table 1:** Social/sexual filters for constructing a contact network.

	For patients1 and 2:
Filter 1	*If (maximum_age_range<|age_1_−age_2_|) connection = 0*
Filter 2	*Rule a: If (r_1_ is not equal to r_2_) connection = 0* *Rule b: If(r_1_ = r_2_ = “Heterosexual” & g_1_ = g_2_) connection = 0* *Rule c: If ( r_1_* = “*Blood products*” or *r_2_* = “*Blood products*”*) connection = 0*
Filter 3	*If (t_1_ is older than s_2_) connection = 0* *If (t_2_ is older than s_1_) connection = 0*

Rules for social/sexual filters. gender (*g*), risk group (*r*), therapy date (*t*), estimated seroconversion date (*s*).

### Analyzing characteristics of the contact network

**Figure 2:**
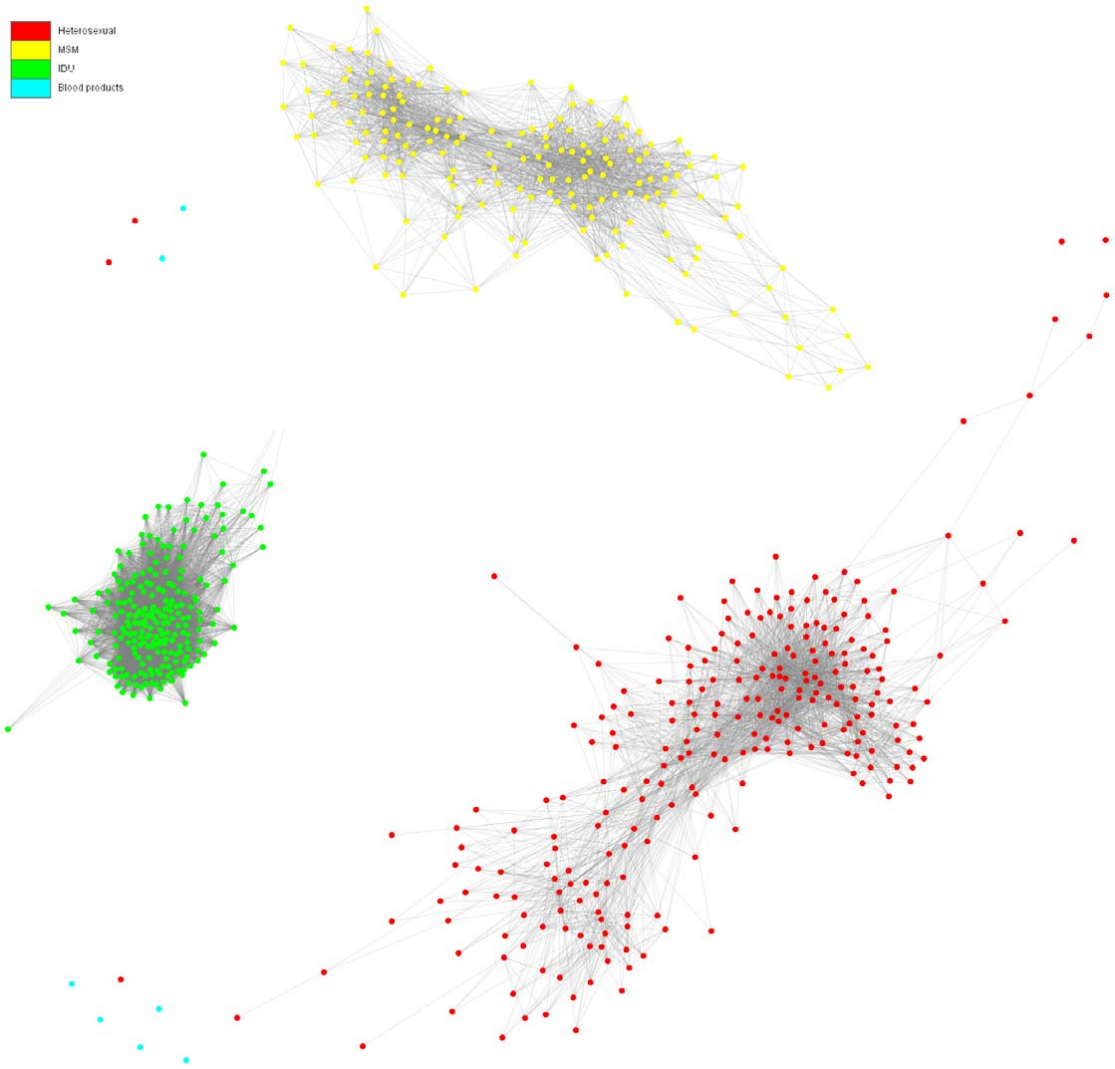
The contact network. Visualization of the contact network consisting of three sub-networks corresponding to the major HIV-1 transmission risk groups: MSM (yellow), Heterosexual (red), and IDU (green).

**Figure 3:**
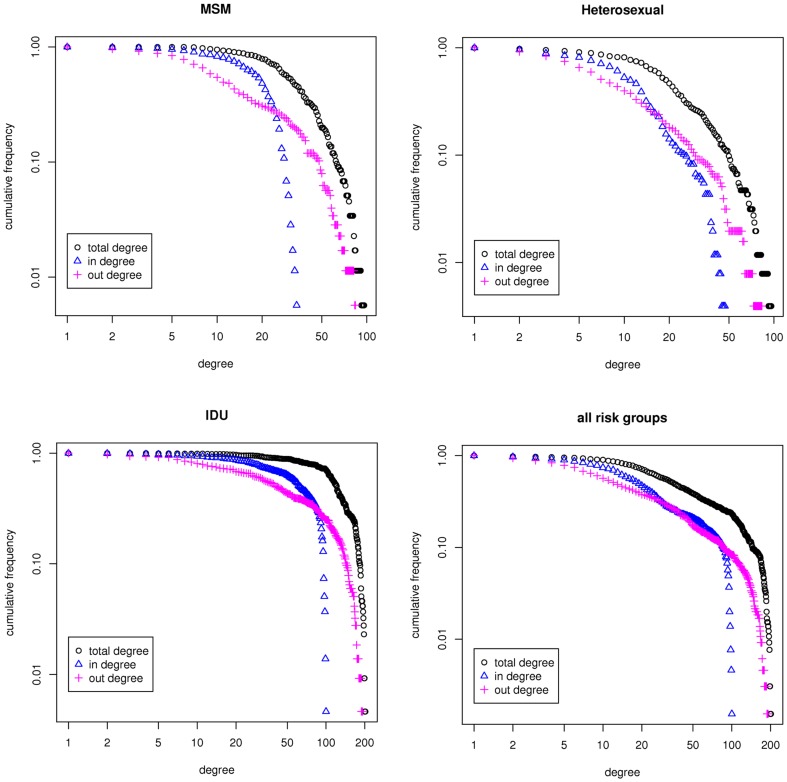
Degree distributions of the contact network. The cumulative total- (black), in- (blue), and out-degree (pink) distributions for the entire network (all risk groups), MSM, Heterosexual, and IDU risk groups plotted in log-log scale.

To analyse the inferred networks we fist visualized the networks and plotted the degree distributions. Figure 2 shows the network for the entire population that consists of three sub-networks corresponding to the major HIV-1 transmission risk groups (MSM, heterosexual, IDU). There were a few patients with “blood product” mode of infection which were isolated from other risk groups. We analyzed the degree distribution of the network as a whole (i.e., for all risk groups) and the degree distribution of each sub-network separately. The cumulative degree distributions of the contact networks of the total-, in- and out-degrees are plotted (log scale) and shown in Figure 3. In-degree is the number of incoming edges to a node and out-degree is the number of outgoing edges from a node. The total degree is the sum of in- and out-degrees.

The degree distributions presented in Figure 3 are based on social and demographical information and are intermediate results before incorporating the genetic data. From the distributions however, one can see that the degree of highly connected patients in IDU is significantly higher than those in MSM and patients acquiring infection through heterosexual contacts. To further investigate the structural differences between networks of the three risk groups, we measured additional network properties including fraction of removed edges, average degree, average path length, global and local clustering coefficients and assortativity, (Table 2).

**Table 2:** Properties of the contact network.

	MSM	Heterosexual	IDU	All risk groups
fraction of removed edges	80.4%	91.2%	45.7%	91.3%
average degree	34.7	22.30	117.1	56.7
average path length	2.16	2.83	1.48	2.20
clustering coefficient (global)	0.59	0.00	0.76	0.71
clustering coefficient (local)	0.70	0.00	0.82	0.47
assortativity (degree)	0.04	−0.20	−0.11	0.45

The percentage of removed edges from the MSM and heterosexual networks is almost twice as the percentage of removed edges from the IDU network. This implies that the MSM and heterosexual contact networks are sparser than the IDU and although the same filters were applied to all risk groups, the nodes in the IDU contact network remains more connected and the network structure is more compact. These observations together with the discrepancies in the degree distributions (Figure 3) and measurements in Table 2 implies that there are structural differences in the contact networks and therefore HIV-1 transmission dynamics between the IDU, MSM and heterosexual populations. The higher degree in the IDU population can be understood from the fact that the IDU was one of the first risk groups affected by the HIV epidemic in Northern Italy and had the highest risk of HIV infection in 1985 [28].Moreover, needle sharing among IDU has a much higher probability of transmission per single act and therefore it is plausible that, besides the differences in trend over time and access to treatment over time regarding the epidemics among the different risk groups, the mode of transmission within IDU by itself might also have contributed to the observed higher degree of distribution. The heterosexual population has a bipartite contact network and therefore the clustering coefficients are zero. Bipartite networks are representative of heterosexual contact networks for *sexually transmitted diseases* (*STDs*) such as HIV/AIDS, since the infection only transmits between males and females and not between individuals with the same gender [29].

**Figure 4:**
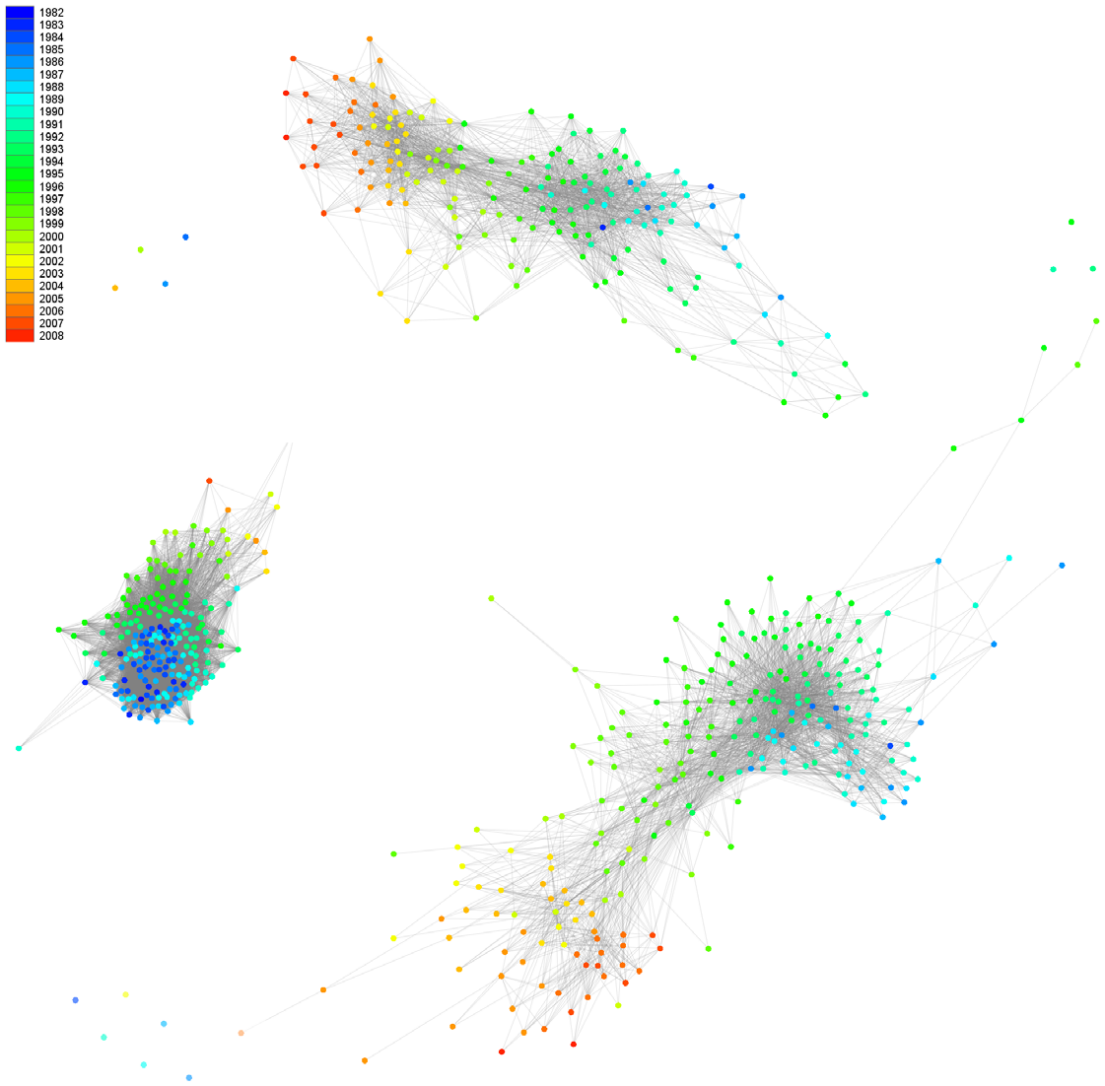
The inferred contact network coloured based on estimated year of seroconversion. The colouring trend in the patient's estimated seroconversion year, ranging from 1982 (blue) to 2008 (red).

We used community detecting methods based on the leading eigenvector of the community matrix to identify community structures in the network [30]. The method helps to identify parts of a network where nodes are densely connected to each other but are sparsely connected to other nodes in the network. The results confirmed the existence of two major communities in the MSM and Heterosexual risk groups (Figure S1). We explain the appearance of these communities from an epidemiological point of view. Formation of communities in a network is due to a local increase in the connectivity between nodes in some parts of the network. Knowing that the connectivity of patients within a community is higher than between communities suggests that people residing in one community had a higher possibility of having contacts and infecting each other. To explore the possible reasons of a higher chance of having infection transmission events between people residing in one community, we mapped the patient's estimated seroconversion years to colour codes from cyan to red. An interesting trend was observed suggesting that the first community (blue to green) contains patients who were infected from 1980 to the late 1990s, while the second community (yellow to red) contains patients who were infected more recently, after the year 2000 (Figure 4). The temporal separation of the communities may reflect the influence of the introduction of more potent and effective anti-retroviral therapies during the second half of the 90 s [31]. The observed trend in the estimated seroconversion year also showed that the HIV-1 incidence in the IDU population decreased over time after the late 80 s (see Figure 4). This is inline the observed decrease in spreading of HIV among the IDU population in Italy after the 80 s as reported by Rezza et al. [32]. However, the trend of HIV infections through different modes of transmissions in our data set (see Figure S2) did not necessarily respect the overall Italian trends [33], [34] and a more representative sample is needed if we want to extend the results from the county/regional to the national scale.

**Figure 5:**
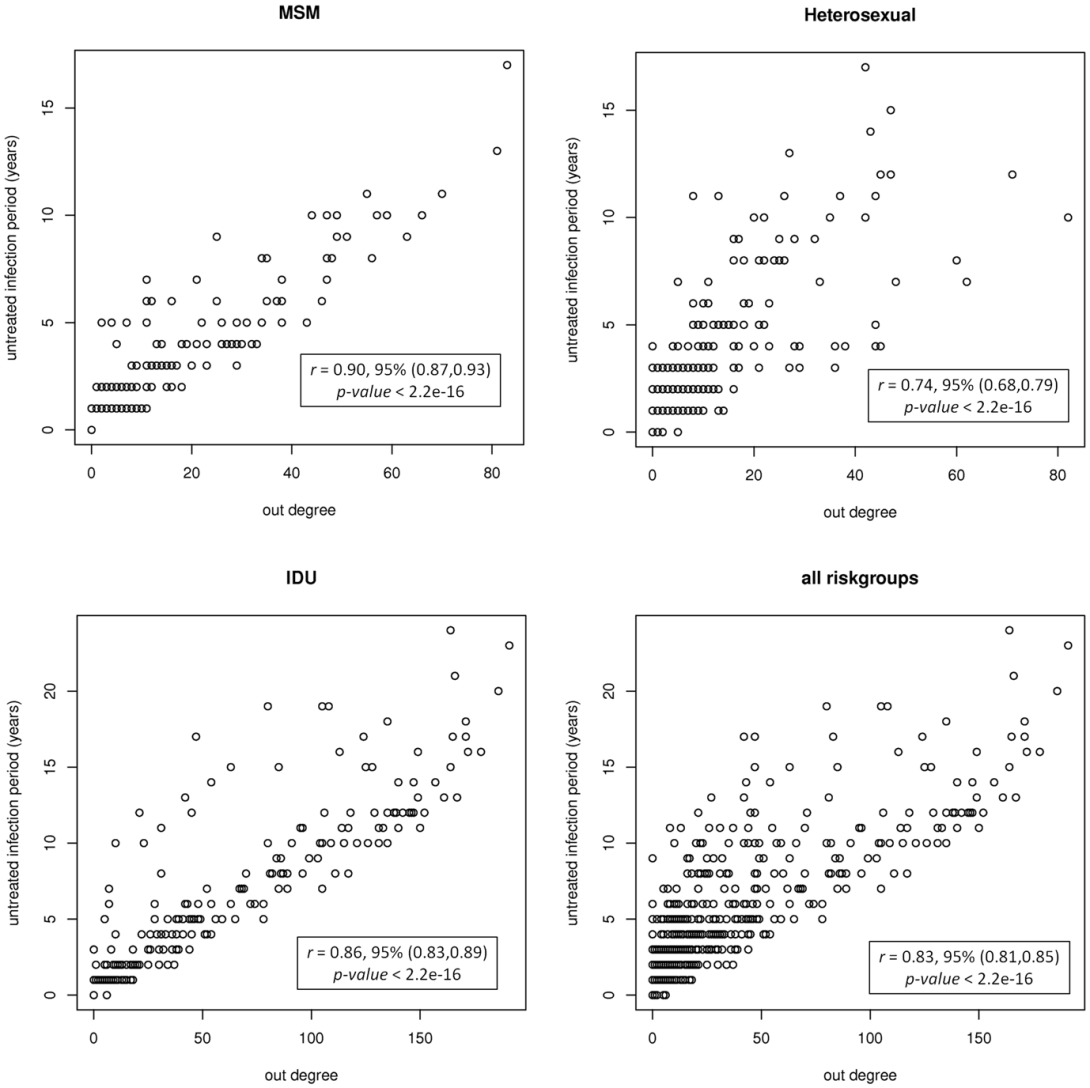
Untreated infection period (UIP) versus out-degree. *UIP* vs. the out-going degree of nodes in the MSM, Heterosexual, IDU and all risk groups populations. The Pearson's correlation coefficients, 95% confidence intervals and p-values are depicted on each graph.

Next we studied the relationship between the untreated infection period and the connectivity of the patients in the network. For that we defined an untreated infection period (*UIP*) for each patient which is computed by:
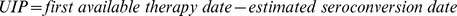

*UIP* is the period that the patient was infected but had not started antiretroviral therapy yet (either because of being unaware of infection or not fulfilling the immuno-virological criteria to be eligible for treatment or not willing to be treated). We detect a correlation between the untreated infection period and the number of out-going edges from a node (out-degree) in the network. The correlation is strongest for the MSM population with a high statistical significance (*r* = 0.90, 95% confidence interval, CI (0.87, 0.93), *p-value*<2.2e-16), where *r* is the Pearson's product-moment correlation. The correlation was less strong but still highly significant for the heterosexual contacts (*r* = 0.74, 95% CI (0.68, 0.79), *p-value*<2.2e-16), IDU (*r* = 0.86, 95% CI (0.83, 0.89), *p-value*<2.2e-16) and the overall population (*r* = 0.83, 95% CI (0.81, 0.85), *p-value*<2.2e-16). The *UIP* versus the out-degree of nodes is plotted in Figure 5 and one can clearly see that nodes with higher out-degree tend to have longer *UIP*s. The inferred networks are direct outcome of the filters we applied. To test the effect of filters on the detected correlations, we rebuilt the networks by each time removing one filter from the filtering process and measured the correlations again. We see that removing the age and risk group filters does not significantly change the correlations. By removing the treatment filter, the correlations decrease but are still statistically significant (data shown in Table S1).

### Constructing the hypothetical transmission networks

**Figure 6:**
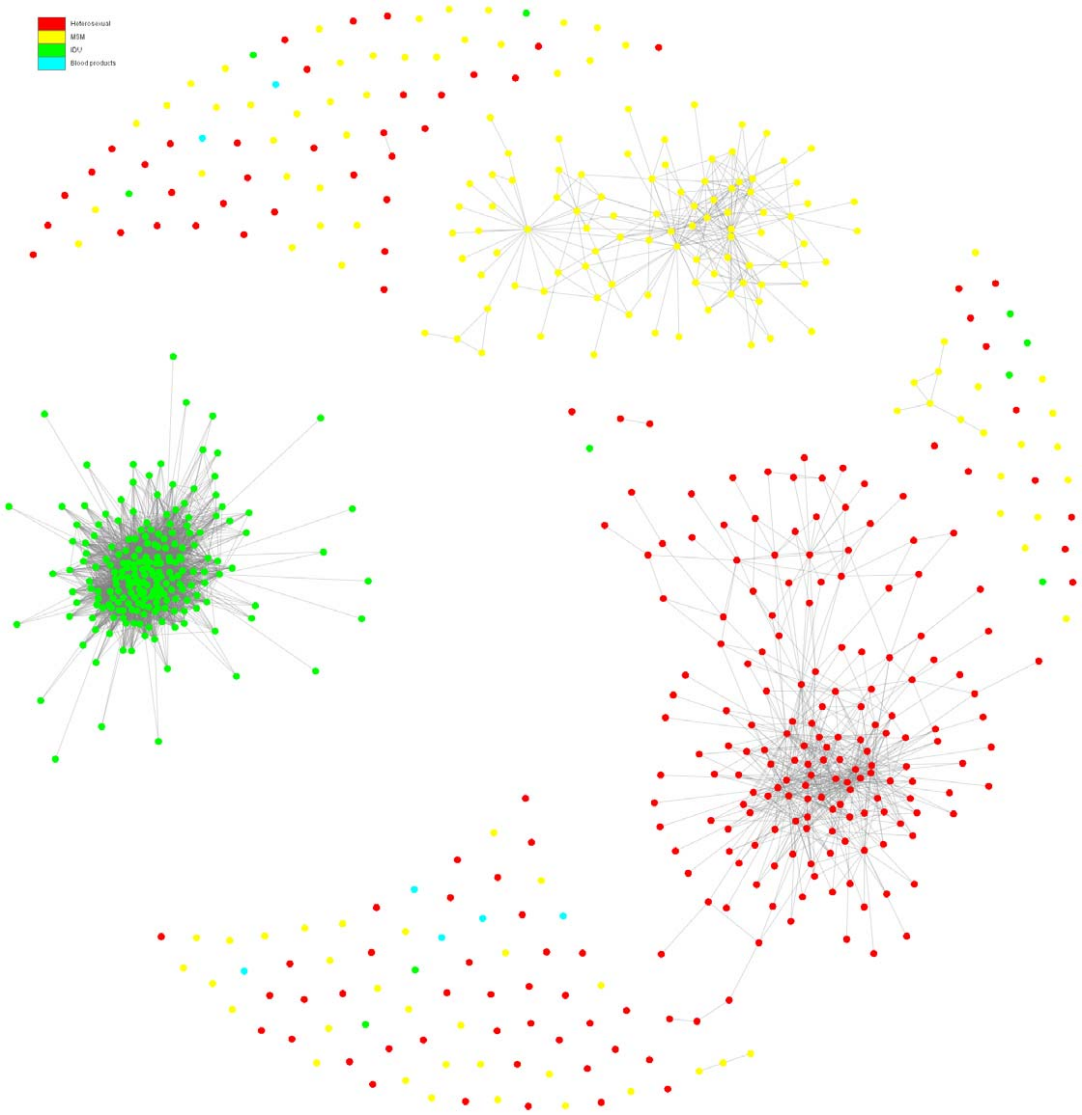
The hypothetical transmission network. The hypothetical transmission network of the entire population obtained from computing the intersection of the contact and the genetic network. Patients are colored based on their risk groups: MSM (yellow), Heterosexual (red), IDU (green) and blood products (cyan).

**Figure 7:**
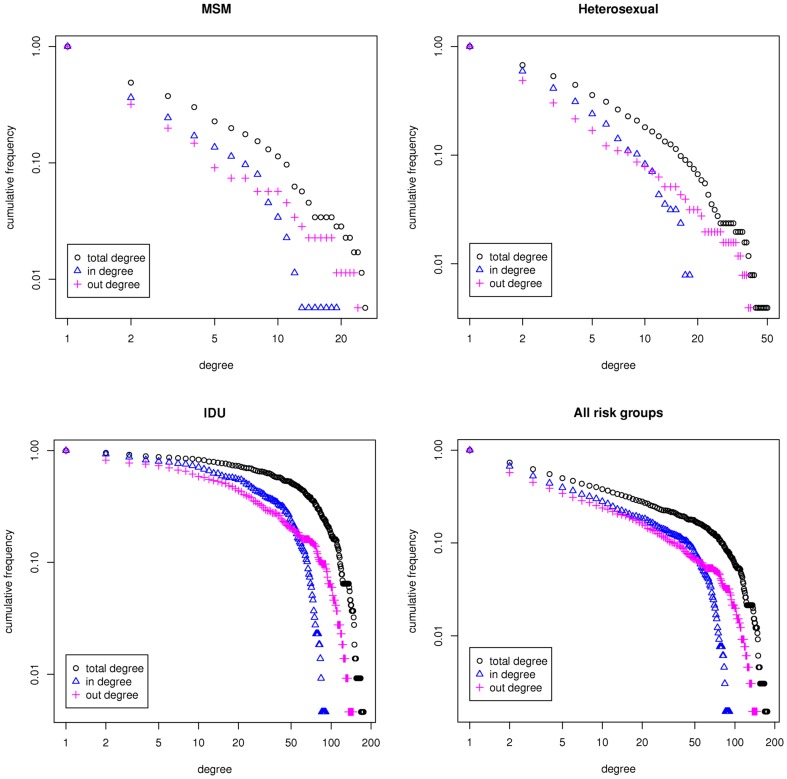
Degree distributions of the hypothetical transmission network. Cumulative total- (black), in- (blue), and out- (pink) degree distributions of the hypothetical transmission network of the MSM, heterosexual, IDU and all risk groups plotted in log-log scale.

To construct a hypothetical transmission network we coupled information from both genetic and epidemiological scales. To this aim, we computed the intersection of the contact network with a genetic network which was obtained from a genetic distance matrix [16], [35]. The genetic distance matrix gives a weighted fully connected network which connects all sequences with each other using their genetic distances as weights (see Dataset S1). The connection between every two nodes with a genetic distance higher than a certain threshold was removed from the network. We used the threshold value of 0.04 nucleotide substitutions per site and derived a genetic network (See Figure S3 and Figure S4). The threshold of 0.04 corresponds to the 15th percentile of the overall distance distribution measured through the phylogenetic tree. The sense is that all retained links include sequences that are closer than the 85^th^ percentile of the all pairwise comparisons (see [17] for a discussion on the optimal threshold). Additionally, we measured the fraction of removed edges from the genetic network by varying this parameter in a range from 0.02 (1^st^ percentile) to 0.05 (35^th^ percentile). We observed that by increasing the threshold value, the percentage of removed edges gradually decreases for the MSM. But, for the heterosexual, IDU and all risk groups the percentages drop under 50% for threshold value 0.05 (Table S2). Subsequently, the genetic network was overlaid with the contact network and the intersection network was computed. The resulting social-genetic intersection network, as a hypothetical transmission network, satisfied both genetic and epidemiological criteria for transmission events. Figure 6 shows the hypothetical transmission network of the entire population. To analyse the characteristics of the inferred network, we plotted the degree distributions (Figure 7) and measured the network properties presented in Table 3.

**Table 3:** Properties of the hypothetical transmission network.

	MSM	Heterosexual	IDU	All risk groups
fraction of removed edges	98.1%	98.0%	74.4%	96.7%
average degree	3.32	4.86	55.30	21.10
average path length	2.86	3.27	1.78	2.22
clustering coefficient (global)	0.36	0.00	0.60	0.59
clustering coefficient (local)	0.50	0.00	0.74	0.45
assortativity (degree)	−0.07	−0.17	−0.22	0.11

In Figure 7, the cumulative degree distributions of the hypothetical transmission networks for the MSM, heterosexual, IDU and for all risk groups are shown. For the MSM and heterosexual populations, the cumulative out-degree distributions were fitted to a straight line, in log-scale, with slopes equal to 2.65±0.43 and 1.88±0.31. Fitting to a straight line in a log-log scale suggests that the degree distribution follows a power-law with a scaling factor equal to the slope [10], [11]. To ensure the fit to the power-law distribution we performed a statistical test, using maximum-likelihood fitting methods with goodness-of-fit tests based on the Kolmogorov-Smirnov statistic [10]. We followed the procedure proposed by Newman et al. (2007) [11] to test for power-law distribution of the data. The method uses maximum likelihood estimators for fitting the power-law distribution to the data, along with the goodness-of-fit based approach to estimate the lower cutoff for the scaling region. The uncertainty in the fitted parameters was estimated using a function that implements the nonparametric approach for estimating the uncertainty in the estimated parameters for the power-law fit. To calculate the p-value for the fitted power-law model, we use a function that implements the Kolmogorov-Smirnov test (which computes a *p*-value for the estimated power-law fit to the data) for the power-law model. If the resulting p-value is greater than 0.1 the power law is a plausible hypothesis for the data, otherwise it is rejected (See Table 4).

**Table 4:** Basic parameters of the data and the power law fit.

quantity	n	Degree	Data			Power law (p)		goodness-of-fit p-value
			*<x>*	σ	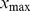	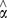	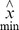	
MSM	176	Total in out	3.32 1.66 1.66	5.62 3.05 3.58	27 18 27	1.82 (0.54) 2.09 (0.38) 2.65 (0.43)	11 (1.80) 2 (1.26) 5 (1.61)	0.0040 0.0590 **0.1730**
Heterosexual	255	Total In out	4.86 2.43 2.43	7.68 3.56 5.67	49 17 39	3.50 (0.61) 2.50 (0.48) 1.88 (0.31)	18 (4.78) 4 (1.71) 2 (1.87)	**0.6130** 0.0030 **0.1020**
IDU	217	Total in out	55.30 27.65 27.65	43.43 23.90 33.54	175 90 146	3.50 (0.12) 3.50 (0.31) 1.96 (0.47)	69 (5.23) 42 (6.02) 15 (12.37)	0.0170 0.0000 0.0000
All risk groups	655	Total in out	21.10 10.55 10.55	35.15 18.47 23.08	175 90 146	3.5 (0.82) 1.6 (0.51) 2.0 (0.29)	69 (27.84) 5 (3.91) 14 (7.24)	0.0160 0.0000 0.0000

Basic parameters of the data (total-, in- and out-degree distributions of the MSM, heterosexual, IDU and all risk groups), along with their power-law fits and the corresponding p-value. Goodness-of-fit tests compare the observed data to the hypothesized power-law distribution. If the resulting p-value is greater than 0.1, power-law is plausible for the data (statistically significant values are denoted in bold).

Then we performed statistical tests (via a likelihood ratio test) to compare the power-law again alternative (Exponential and Poisson) distributions for the data. For each alternative distribution, we computed a likelihood ratio shown in Table 5. If the calculated likelihood ratio is significantly different from zero, then its sign indicates whether the alternative is favored over the power-law model or not. The statistical tests results and positive likelihood ratios show that the MSM out-degree distribution is a good fit to the power law model in comparison to Exponential and Possion distributions.

**Table 5:** Test of power law behavior in the data and likelihood ratios of alternative distributions.

	Power law (p-value)	Poisson		Exponential		Support for power law
		LR	p-value	LR	p-value	
MSM (out-degree)	**0.1730**	2.31	**0.02**	0.35	0.72	good
Heterosexual (total-degree)	**0.6130**	4.08	**<0.01**	−2.67	**0.01**	moderate
Heterosexual (in-degree)	**0.1020**	3.28	**<0.01**	1.85	**0.06**	good

For each degree distribution we give a p-value for the fit to the power-law model and likelihood ratios (*LR*) for the alternatives. We also quote p-values for the significance of each of the likelihood ratio tests. Significant p-values are denoted in bold. Positive values of the likelihood ratios indicate that the power-law model is favored over the alternative. The final column of the table lists the judgment of the statistical support for the power-law hypothesis for each distribution. “Moderate” indicates that the power-law is a good fit but there are other plausible alternatives as well; “good” indicates that the power-law is a good fit and that none of the alternatives considered is plausible.

### Transmission network and phylogenetic clusters

We compared the inferred transmission network with a set of genetic clusters obtained through phylogenetic analysis of the corresponding viral sequences (see Materials and Methods and Figure S5). A total of 61 clusters (from size 2 to 52) were identified, where 39% of all patients were included in these clusters (see Figure S6 for the cluster size distribution). Nodes, representing individual viral isolates, residing in the same cluster are identified to be genetically close and therefore, possibly transmitted the virus to each other. For every two nodes in a same genetic cluster we tested if they were connected (directly or indirectly) in the transmission network. The percentage of genetically close nodes that were connected in the transmission network was 37% for MSM, 55% for heterosexual, and 95% for IDU. The high percentage of genetically close nodes in the IDU population also supports the idea that the needle sharing does play an important node in the transmission of HIV in the resulting contact network.

### Factors associated with super-spreaders

High out-degree nodes in the network have a higher probability of out-spreading the virus to more contacts. In a population these nodes can play the role of super-spreaders with lot of connections [36]–[39]. In Table 6, we report the results of a multivariable linear regression analysis conducted to identify factors associated with super-spreaders or higher out-degree nodes in the network. In all populations a longer untreated infection period and a higher number of incoming links were associated with super-spreaders. The risk of being a super-spreader was also associated with a higher viral load and an older age in the MSM population. The risk in males was higher than females in the heterosexual population and in all risk groups. We also performed a univariable regression analysis to identify the independent effect of covariates with respect to super-spreaders (See Figure S7).

**Table 6:** Factors associated with out-degree nodes.

Factor/risk group	MSM			Heterosexual			IDU			All risk groups		
	Coef	Std	P value	Coef	Std	P value	Coef	Std	P value	Coef	Std	P value
**Age (years)**	0.02	0.01	0.0078	0.01	0.01	0.7788	0.03	0.00	<0.0001	0.02	0.00	<0.0001
**Viral load (copies/ml)**	0.02	0.06	0.7450	−0.03	0.04	0.5242	−0.06	0.01	0.0001	−0.08	0.01	<0.0001
**UIP (years)**	0.22	0.01	<0.0001	0.24	0.01	<0.0001	0.13	0.00	<0.0001	0.17	0.00	<0.0001
**Gender (Male/Female)**	-	-	-	0.39	0.10	0.0001	0.09	0.03	0.0044	0.31	0.03	<0.0001
**In-degree**	0.12	0.01	<0.0001	0.15	0.01	<0.0001	0.01	0.00	<0.0001	0.03	0.00	<0.0001

Results of a multi-variable regression analysis showing the factors associated with high out-degree nodes. The out-degree is the dependent variable in the analysis, and age, viral load, UIP, gender, and In-degree are independent variables.

### Comparison with random networks

To compare the hypothetical transmission networks with random graphs, we generated random networks of the same size (nodes and edges) as the inferred transmission networks for each population (MSM, heterosexual, IDU and all risk groups). For this, we used the fraction of remaining edges in each network, as a probability to generate an edge in the random network. Table 7 compares the properties of the inferred transmission networks with random networks. One can see that the inferred networks are different from random networks of their own size by having lower average path lengths, higher clustering coefficients and higher assortativity coefficients.

**Table 7:** Properties of the hypothetical transmission network against random networks.

	MSM		Heterosexual		IDU		All risk groups	
	Inferred	Randomized	Inferred	Randomized	Inferred	Randomized	Inferred	Randomized
average degree	3.32	3.24	4.86	5.02	55.30	55.35	21.10	21.50
average path length	2.86	4.38	3.27	3.59	1.78	1.74	2.22	2.44
clustering coefficient (global)	0.36	0.02	0.00	0.02	0.60	0.25	0.59	0.032
clustering coefficient (local)	0.50	0.02	0.00	0.01	0.74	0.25	0.45	0.032
assortativity (degree)	−0.07	−0.02	−0.17	0.03	−0.22	−0.02	0.11	<−0.01

Both inferred and randomized networks are of the same size in terms of number of nodes and edges. The properties of the randomized network is an average over the properties of 5 random networks.

## Discussion

A new method for inferring hypothetical HIV-1 transmission networks is introduced using information from both genetic and epidemiological scales. This study constitutes, to the best of our knowledge, the first attempt to combine social and genetic data to characterise transmission networks for HIV-1. We propose a new filter-reduction method for network construction and used it to build a network of HIV-1 sequences based on their connected social and demographical information. To characterise the hypothetical transmission networks we compute the intersection of the social network with the genetic network obtained from the genetic distance matrix of Italian patients. Standard network approaches consider a predefined network structure with certain parameter values to build a network, such as scale-free structure with an exponent in the range of 1.5 to 2.0 for the MSM population in HIV transmission [5], [6]. The main advantage of the method presented here is that it does not require any pre-assumption on the network structure. The network structure itself is an emergent characteristic of our approach. The power-law distribution for the MSM and heterosexual out-degree distributions yields a scale-free structure for these networks with exponents equal to 2.65 and 1.88. This means that the structure of the hypothetical transmission network for the MSM and heterosexual population is heterogeneous, consisting of a majority of ‘peripheral nodes’ that have only a few sexual interactions and a minority of ‘hub nodes’ that have many sexual interactions. This finding is in line with the results obtained from analysis of the degree distribution of HIV transmission networks for the MSM population in the UK [23].

Interestingly, we uncover a positive correlation between the duration of untreated infection periods and the out-degree of the nodes in the network. This important finding may be explained by the fact that untreated individuals have higher viral loads and are therefore more infectious; moreover not being on therapy is generally associated to a higher probability of not being diagnosed or not being compliant to treatment and prevention messages conveyed by health care providers. This finding underscores the importance of case finding, early diagnosis and anticipated antiretroviral treatment as tools to prevent HIV-1 transmission and spread [40], [41].

The delay between the median estimated seroconversion and the start of genotyping may have caused the older half of infections to be a bias sample, as in the pre-HAART (highly active antiretroviral therapy) era when only the slow progressors survived to be genotyped later. To investigate this effect, we perform the analysis on a subset of recent infections, by only considering instances with first positive test after 1998 calendar year. There were 202 patients with a recent infection in the data in which 79 were MSM, 99 were Heterosexual, 24 were IDU. The correlation between the untreated infection period and the out-degree of nodes in the contact network still holds (Figure S8). However, the degree distributions of the transmission network did not pass the statistical test for fit to a power-law. The number of 202 recent infections in our current dataset is relatively a small sample. Doing the analysis on recent infections is worthwhile but requires having access to recently collected data, which will definitely be considered in our future studies.

Super-spreaders are highly infectious individuals with a high viral load and a high rate of partner change [36], [42]. Identifying and controlling these super-spreaders is crucial for stopping the spread of disease in a population [43], [44]. The identified factors associated with super-spreaders highlighted in the results section could help to achieve this goal. The identified correlation presented in this paper also suggests the association of hubs in the network (super-spreaders) with not being on antiretroviral treatment for longer periods. The stages of infection between the seroconversion, the detection of the infection, and the initiation of therapy are crucial in driving the transmission epidemics. Individuals who do not test regularly and have a risky sexual behaviour can more easily become hubs or super-spreaders, along with those who do not initiate a therapy early after the first positive test and do not change at risk behaviours. The fact that, in this study, networks' hubs were those with a longer untreated period confirms this hypothesis. Until recently, the initiation of antiretroviral treatment has not been decided by a transmission prevention policy, but rather by considering patient's immunological conditions [HIV-AIDS treatment 2011 guidelines: http://www.aidsinfo.nih.gov/contentfiles/adultandadolescentgl.pdf]. Our observation, along with data presented from recent clinical studies [40], [45], strongly suggests that early treatment should be considered in order to prevent transmission, although the cost-benefit of such a strategy must be further assessed in different populations and epidemiological scenarios.

The transmission of HIV drug resistance is another important clinical and epidemiological concern which induces treatment failure. Approximately 10% of newly diagnosed patients with HIV-1 infection in Europe are infected with a drug resistant virus [46], [47]. Therefore, there is an urgent need for prevention strategies in order to block the transmission of drug resistant virus. Characterisation of the HIV transmission networks proposed in this paper is a first step that can facilitate the investigations on the transmission of viral drug resistance.

In this study we have limited ourselves to transmission within the three main risk groups, omitting transmission between risk groups which are also observed in the phylogenetic analysis [17]. The reason for that was having no access to reliable social and behavioral data to include transmission between risk groups and we will consider extending our current study in that direction upon availability of the required data.

We believe that the new approach presented here for inferring transmission networks can have important repercussions in the design of intervention for disease control not only for HIV, but potentially for a wide range of viruses and emerging pathogens.

## Materials and Methods

In this study, we combined information from both genetic (derived from HIV-1 RNA sequences) and epidemiological scales to characterize a transmission network of the HIV-1 epidemic in central Italy. The study population included HIV-1 infected patients, with viral genotyping between 1997 and 2009, enrolled and followed up at the Clinic of Infectious Diseases of the Catholic University of the Sacred Heart in Rome, Italy. Inclusion criteria were to have at least one viral genotype sequence performed for each patient, allowing multiple observations for patients with more than a viral genotype available. We applied a novel filter-reduction method to infer a network of HIV-1 sequences based on the corresponding patient's epidemiological information, obtaining a potential contact network. The method is based on real patient data and no pre-assumptions are made on the network structure. To characterize the transmission network of HIV-1, the intersection of the contact network with a genetic network based on a genetic distance matrix was computed.

### The Data

HIV-1 RNA sequences from a region-wide cohort study of HIV-1-infected people in Rome and Lazio region, Italy, were used [The database is a part of the three national HIV data cohort in Italy: ARCA (www.hivarca.net), Icona (http://www.fondazioneicona.org), and Master (http://www.mastercohort.it)]. The viral sequence information encompassed the HIV *pol* gene region, covering the whole protease and most of the reverse transcriptase gene (at least the first 1–250 amino acids). Sequence data was annotated with corresponding patient's demographics and treatment information, including: sequence id (numeric), viral subtype, sequence calendar year (numeric), patient's gender (male/female), age (numeric), mode of HIV transmission (MSM, heterosexual, IDU, blood products, other/unknown), country of origin (Italian/non-Italian/unknown), ART status (ART-experienced/ART-naive), seroconversion year (median time between last HIV-1 negative test date and first HIV-1 positive test date), calendar year of first HIV positive test and of first available antiretroviral therapy (numeric), plasma HIV-RNA load (numeric) at viral sequencing time, presence of resistance mutations for nucleoside-tide/non-nucleoside reverse transcriptase inhibitors and protease inhibitors in the HIV-1 sequence (binary). The unknown/other risk group members were excluded from the analysis. In the case of missing values for the last negative test date, in order to estimate the seroconversion we take the first positive test date minus one year which is the average time difference between the estimated seroconversion date and first positive test in the data. For a number of patients in the dataset, multiple sequences were recorded at different time points, but we only considered the earliest sequence per patient for social/epidemiological analysis. The sequence data was used for phylogenetic analysis and subsequent inference of transmission clusters, while the annotated demographical and treatment information were used for social network construction. The statistics of patient's characteristics are presented in Table 8.

**Table 8:** The statistics of patients characteristics (total n = 655, subtype B patients, excluding entries with unknown risk group).

	Data statistics				Number of unknown/missing data entries
Risk group	22.8% MSM (n = 176)	33.0% heterosexual (n = 255)	28.0% IDU (n = 217)	0.9% blood products (n = 7)	-
Gender	65% male (n = 426)		35.0% females (n = 229)		-
country of origin	84.0% Italian (n = 553)		10.4% non-Italian (n = 68)		5.2% unknown (n = 34)
Antiretroviral therapy	19.3% therapy-naïve (n = 127)		80.7% therapy-experienced (n = 528)		-
	median (IQR)				
Age	48 (43–53) years				-
Estimated seroconversion date	1996 (1993–2000) calendar year				79.0% unknown (n = 517)
Last negative test date	1995 (1991–1999) calendar year				78% unknown (n = 515)
First available positive test date	1995 (1991–2000) calendar year				-
viral genotyping date	2004 (2001–2007) calendar year				0.4% unknown (n = 3)
First available therapy date	1998 (1995–2003) calendar year				-
plasma viral load (At the time of viral genotyping)	4.1 log10 HIV RNA copies/ml (3.5–4.7)				0.4% unknown (n = 3)
time from estimated seroconversion date to the first therapy date	3 (1.25–5) years				79.0% unknown (n = 517)
time from estimated seroconversion date to the first viral sequence date	8 (4–11) years				79.0% unknown (n = 517)

### Phylogenetic analysis

HIV-1 sequences matching the inclusion criteria were aligned using MUSCLE software [48] and the resulting multiple alignments were edited in order to remove drug-resistance associated mutations [IAS-USA list 2010 (http://www.iasusa.org/pub/topics/2010/issue5/156.pdf)] that can lead to a convergent evolution bias in the phylogenetic tree estimation. A phylogenetic tree was then estimated using the maximum likelihood FastTree software [49], assessing node reliability via the built-in Shimodaira-Hasegawa test. Transmission clusters were extracted from the phylogenetic tree using the PhyloPart java application [17]. The PhyloPart uses a depth-first algorithm to extract a crisp partition (i.e. clustering) from an input phylogenetic tree, constraining its search on the comparison between sub-tree (i.e. potential clusters) and whole-tree patristic distance distributions, plus additional ancillary topologic criteria. When the sub-tree is highly (>90%) supported by bootstrap (or posterior probability or other statistical test), when at least two distinct patients are in the sub-tree, and when the median patristic distance is below a percentile threshold of the whole-tree distance distribution, then a cluster is found. If the depth-first search reaches a leaf node without finding any cluster, then the instance is classified as a singleton. Additionally, a genetic distance matrix was calculated with the MEGA software using the LogDet function [50].

### Filtering process in the filter-reduction method

The filter-reduction method was used to build a contact network from the dataset. Each node in the network represents a viral sequence isolate of HIV-1 obtained from a patient. Starting from an undirected fully-connected network of all patients, a set of social/sexual filters was applied. These filters considered patients' demographical and treatment information. A direct connection between every two nodes that did not satisfy the epidemiological criteria was removed from the network (the percentage of removed edges from the network by applying each filter is presented in Table S3). In what follows the social filters for building the contact network are described in more detail:

- **Filter 1:** The age filter indicates the maximum age range for an individual to be socially or sexually interactive with another individual. If the age difference between two patients exceeds the maximum age range the direct connection between them is filtered. The age difference is a free parameter and can be changed. We used a value of 10 years for this parameter based on a study on age-disparate and intergenerational sex in South Africa [51]. We also perfrmend a sensitivity analysis on this parameter by varying the value between 2 to 20 years (data shown in Table S4).
- **Filter 2:** This filter considers the patient's gender (*g*) and risk group (*r*). Three rules are implemented: *Rule a*: the connection between patients from different risk groups is filtered, this results in creation of three separate sub-networks corresponding to the major HIV transmission risk groups (MSM, Heterosexual, and IDU). *Rule b:* for the heterosexual risk group the connection between patients with the same gender is filtered. *Rule c*: The “Blood product” risk groups are isolated from the population, as they were not infected through sexual relationships.
- **Filter 3:** Observational studies suggest that the transmission probability of HIV-1 decreases by 80–98% after a patient starts treatment [52], [53]. This is mainly due to the smaller amount of viral particles in the genital secretions and mucosa after treatment and the behavioural changes in the patients sexual and social habits when they become aware of their disease. Following this observation, we filtered connections to a patient A from any other patient whose therapy initiation date (*t*) predated patient A's estimated seroconversion date (*s*).

### Network visualization

The network visualizations in this article were produced using an in-house developed interactive visualization tool, called “Twilight”, which is based on the igraph software package for complex network research [54]. The layout for all graphs was produced using an implementation of Fruchterman-Reigngold algorithm provided by igraph [55]. A demo of network visualization is shown in Video S1 and more information on Twilight can be found at http://uva.computationalscience.nl.

## Supporting Information

Figure S1 **Communities in the MSM and heterosexual populations.** Two main communities (green and blue) identified in the MSM and heterosexual populations using community structure detecting methods based on the leading eigenvector of the community matrix. The red edges are connecting different communities.
(TIF)

Figure S2 **Prevalence of mode of transmission groups stratified by calendar year in the study population.**
(TIF)

Figure S3 **Visualization of the genetic network.** The genetic network is built based on the genetic distance matrix. There is a link between every two patients in the network if their genetic distance is smaller than the threshold value of 0.04 ns/s. Patients are coloured based on their corresponding risk group: MSM (yellow), heterosexual (red), IDU (green) and blood products (cyan).
(TIF)

Figure S4 **Degree distributions of the genetic network.** Cumulative total- (black), in- (blue), and out-degree (pink) distributions of the genetic network plotted in log-log scale for the MSM, Heterosexual, IDU and all risk groups.
(TIF)

Figure S5 **Phylogenetic tree and genetic clusters.** Phylogenetic tree with the leaves colored as cluster Ids (nodes residing in one genetic cluster have the same cluster Id). The colors have been generated by dividing the RGB spectrum into specific intervals, corresponding to the number of distinct clusters. The red leaves scattered through the whole tree are “singletons” (i.e. unclustered isolates).
(TIFF)

Figure S6 **Genetic clusters size distribution.** Genetic clusters extracted from the phylogenetic tree analysis. A total of 61 clusters (from size 2 to 52) were identified and 39% of all patients were included in these clusters.
(TIF)

Figure S7 **Univariable regression analysis of factors associated with super-spreaders.** Plots of numerical factors (age, viral load, *UIP* and in-degree) versus the out degree of nodes in the MSM, heterosexual, IDU and all risk groups. The correlation coefficients depicted on the graphs show the strength of a linear relationship between independent factors with respect to super-spreaders.
(TIF)

Figure S8 **Untreated infection period (UIP) versus out-degree of recent infections.** *UIP* vs. the out-going degree of nodes in the MSM, Heterosexual, IDU and all risk groups populations, for recent infections in the dataset (instances with first positive test after 1998 calendar year). The Pearson's correlation coefficients, 95% confidence intervals and p-values are depicted on each graph.
(TIF)

Table S1 **Correlation between the UIP and out-degree of the nodes by removing each filter from the filtering process in network construction.** None implies that all filters are applied and none is removed from the filtering process.
(DOC)

Table S2 **Fraction of removed edges from the genetic network using different genetic thresholds.** Each threshold value corresponds to a percentile of the overall distance distribution measured through the phylogenetic tree.
(DOC)

Table S3 **Percentage of edges filtered from the network by applying each different filter and all filters.**
(DOC)

Table S4 **Sensitivity analysis on the “maximum age difference” parameter.**
(DOC)

Dataset S1 **Genetic distance matrix.** Excel file of the measured genetic distance between every two viral sequences in the Italian patient dataset.
(CSV)

Video S1 **Appearance of risk group clusters in a contact network.** The video shows the construction of a contact network and appearance of three clusters corresponding to the three major HIV risk groups (MSM, heterosexual, IDU).
(RAR)
