# Supplementary figures and images for: Combining Epidemiological and Genetic Networks Signifies the Importance of Early Treatment in HIV-1 Transmission
Source: PLoS One. 2012 Sep 28;7(9):e46156. doi: 10.1371/journal.pone.0046156 (PMC3460924; doi:10.1371/journal.pone.0046156)

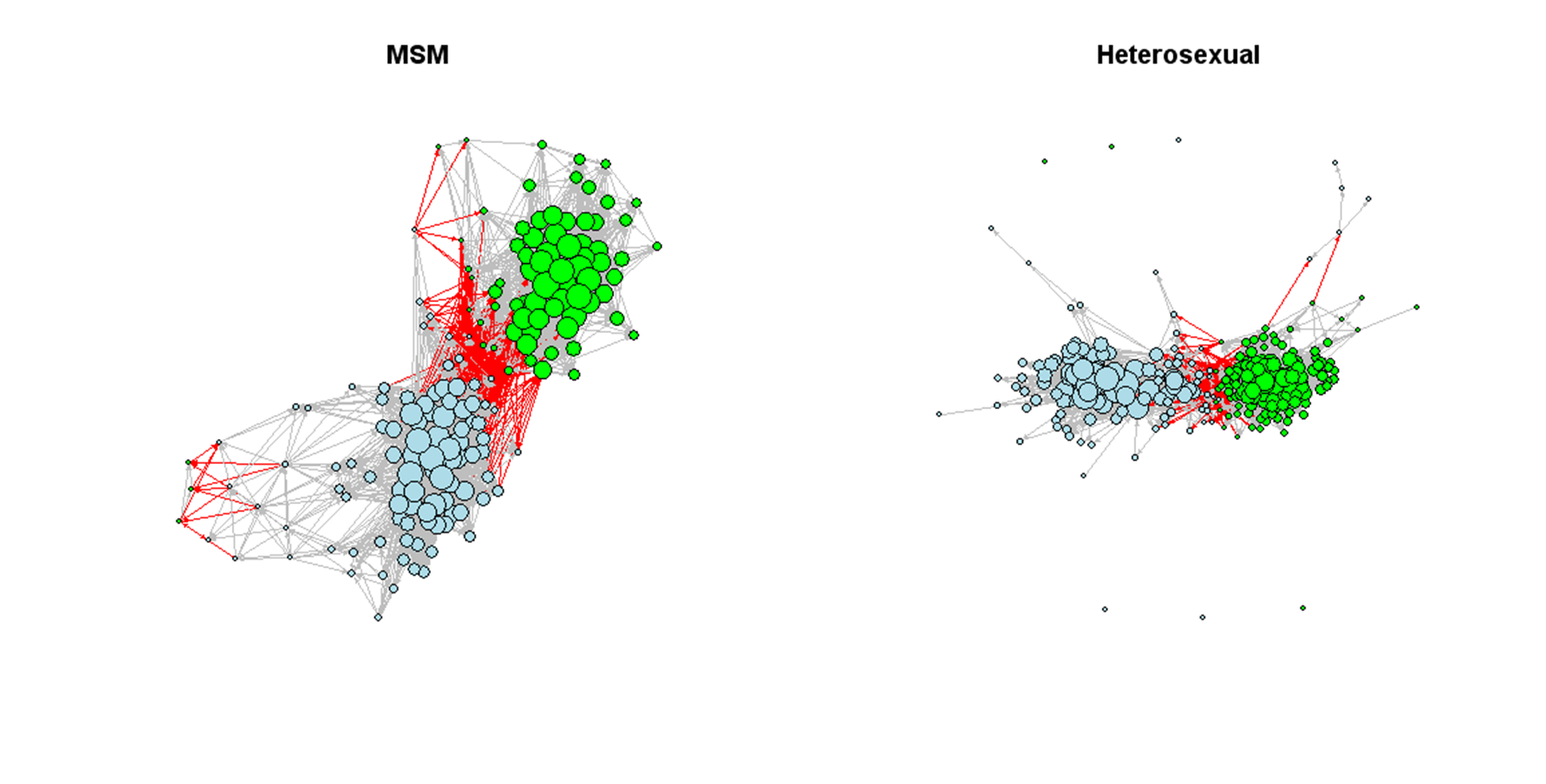

Supplement: Figure S1 — Communities in the MSM and heterosexual populations. Two main communities (green and blue) identified in the MSM and heterosexual populations using community structure detecting methods based on the leading eigenvector of the community matrix. The red edges are connecting different communities. (TIF) [file pone.0046156.s001.tif]

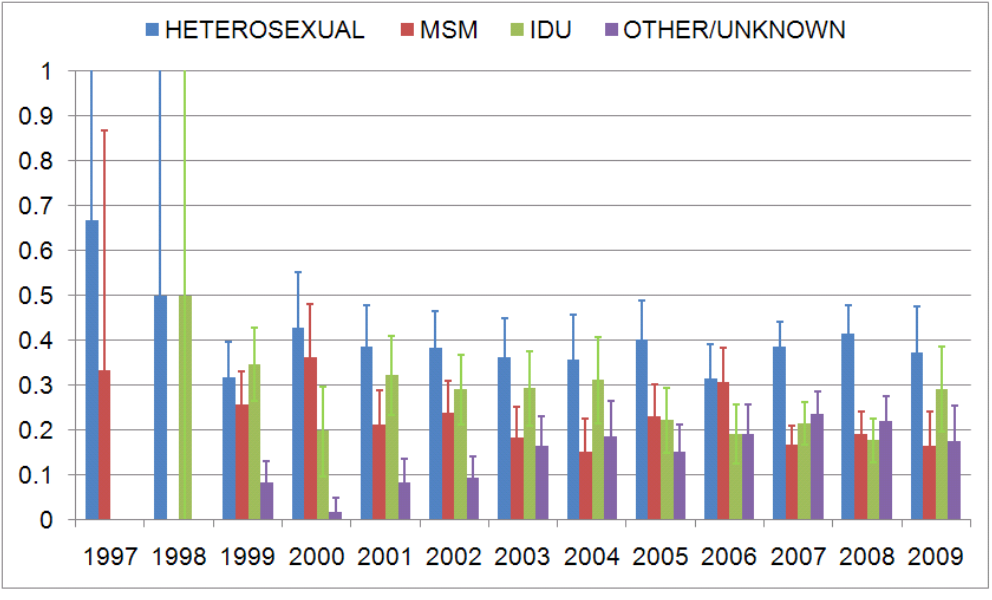

Supplement: Figure S2 — Prevalence of mode of transmission groups stratified by calendar year in the study population. (TIF) [file pone.0046156.s002.tif]

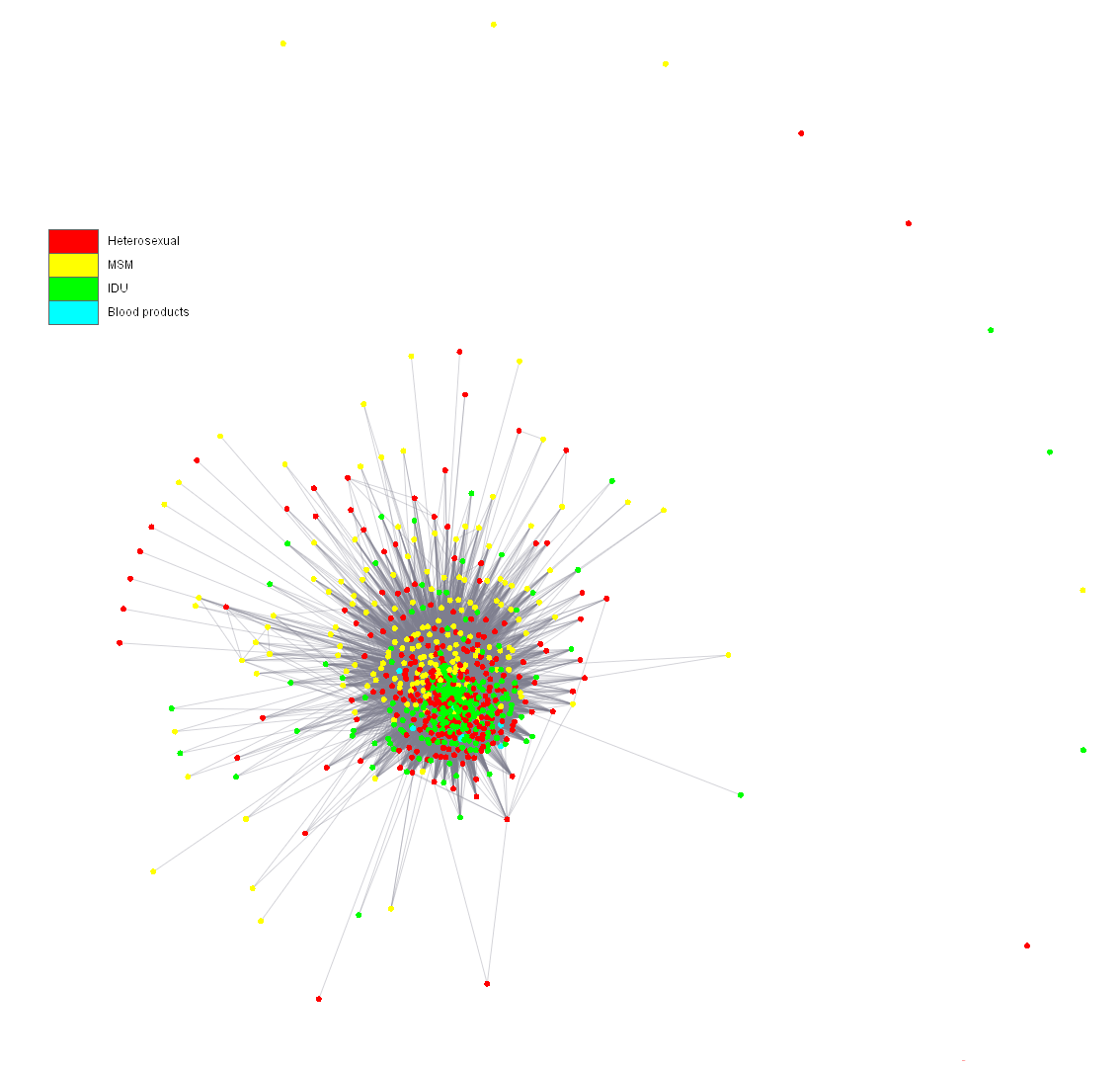

Supplement: Figure S3 — Visualization of the genetic network. The genetic network is built based on the genetic distance matrix. There is a link between every two patients in the network if their genetic distance is smaller than the threshold value of 0.04 ns/s. Patients are coloured based on their corresponding risk group: MSM (yellow), heterosexual (red), IDU (green) and blood products (cyan). (TIF) [file pone.0046156.s003.tif]

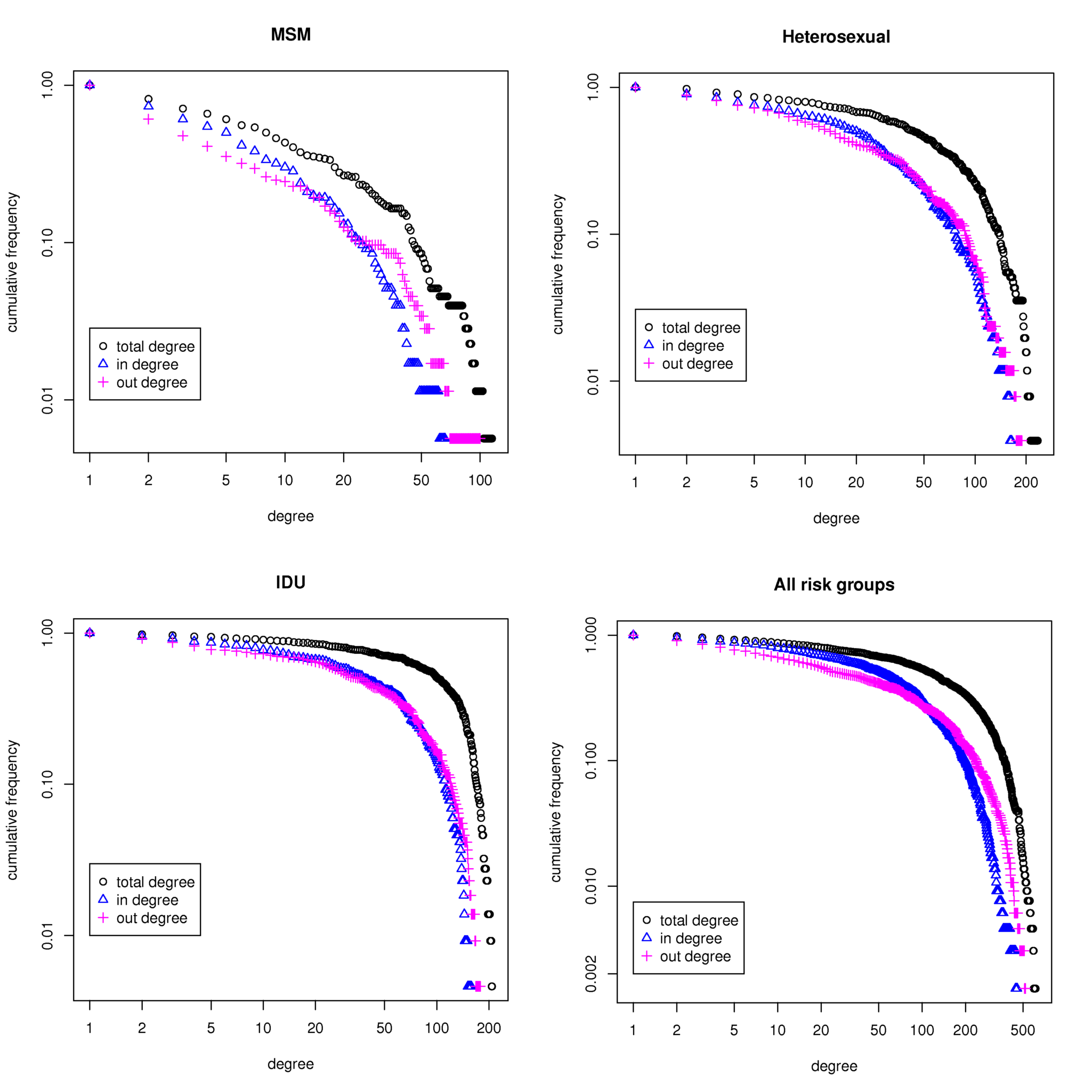

Supplement: Figure S4 — Degree distributions of the genetic network. Cumulative total- (black), in- (blue), and out-degree (pink) distributions of the genetic network plotted in log-log scale for the MSM, Heterosexual, IDU and all risk groups. (TIF) [file pone.0046156.s004.tif]

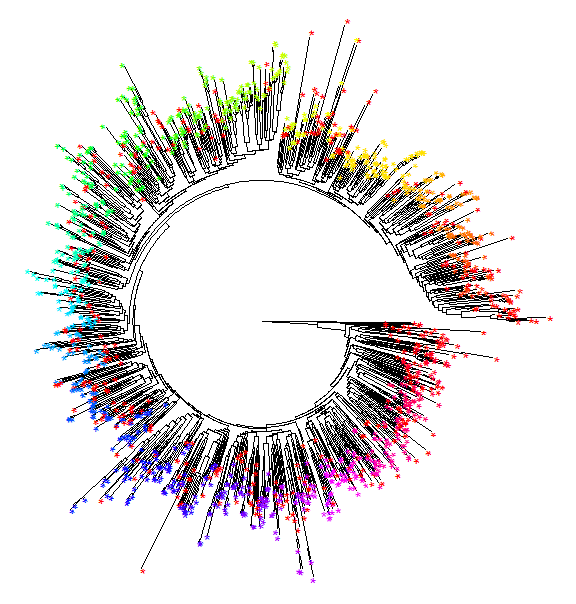

Supplement: Figure S5 — Phylogenetic tree and genetic clusters. Phylogenetic tree with the leaves colored as cluster Ids (nodes residing in one genetic cluster have the same cluster Id). The colors have been generated by dividing the RGB spectrum into specific intervals, corresponding to the number of distinct clusters. The red leaves scattered through the whole tree are “singletons” (i.e. unclustered isolates). (TIFF) [file pone.0046156.s005.tif]

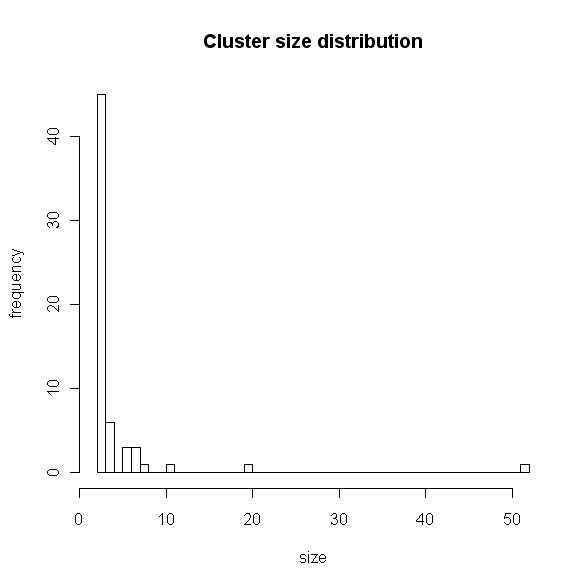

Supplement: Figure S6 — Genetic clusters size distribution. Genetic clusters extracted from the phylogenetic tree analysis. A total of 61 clusters (from size 2 to 52) were identified and 39% of all patients were included in these clusters. (TIF) [file pone.0046156.s006.tif]

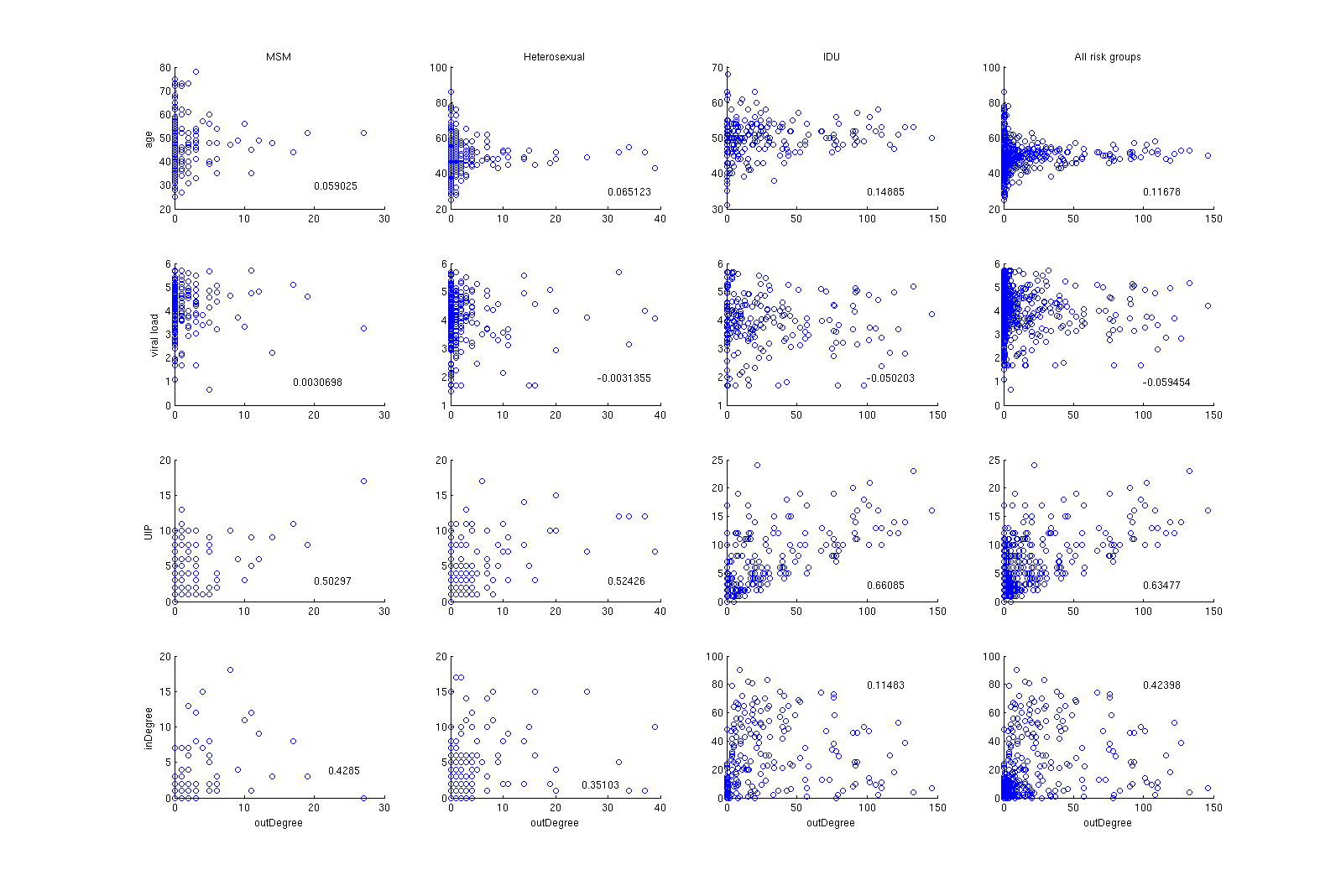

Supplement: Figure S7 — Univariable regression analysis of factors associated with super-spreaders. Plots of numerical factors (age, viral load, UIP and in-degree) versus the out degree of nodes in the MSM, heterosexual, IDU and all risk groups. The correlation coefficients depicted on the graphs show the strength of a linear relationship between independent factors with respect to super-spreaders. (TIF) [file pone.0046156.s007.tif]

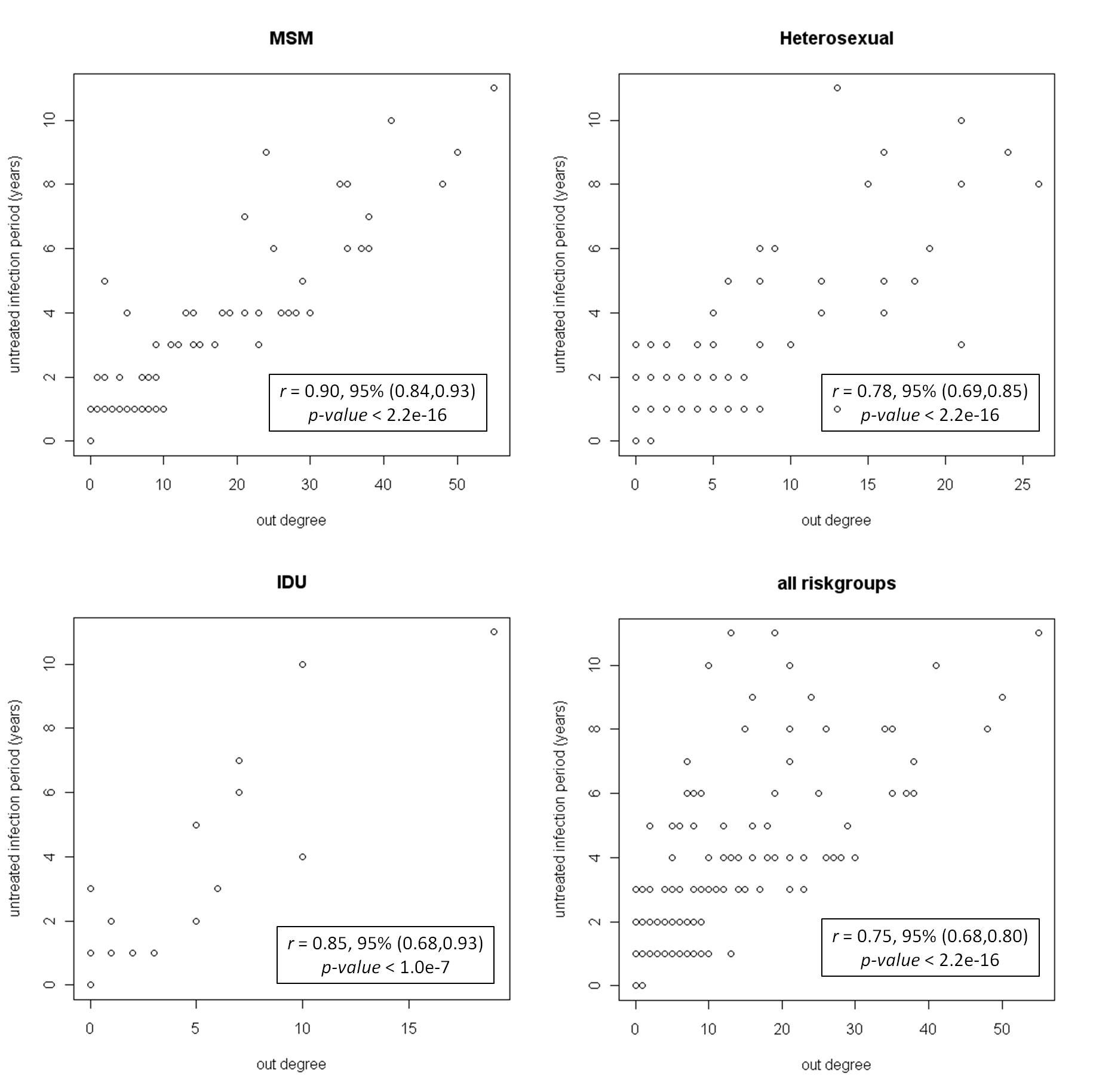

Supplement: Figure S8 — Untreated infection period (UIP) versus out-degree of recent infections. UIP vs. the out-going degree of nodes in the MSM, Heterosexual, IDU and all risk groups populations, for recent infections in the dataset (instances with first positive test after 1998 calendar year). The Pearson's correlation coefficients, 95% confidence intervals and p-values are depicted on each graph. (TIF) [file pone.0046156.s008.tif]
