# Supplementary material for: Combining Epidemiological and Genetic Networks Signifies the Importance of Early Treatment in HIV-1 Transmission
Source: PLoS One. 2012 Sep 28;7(9):e46156. doi: 10.1371/journal.pone.0046156 (PMC3460924; doi:10.1371/journal.pone.0046156)
Supplement: Table S1 — Correlation between the UIP and out-degree of the nodes by removing each filter from the filtering process in network construction. None implies that all filters are applied and none is removed from the filtering process. (DOC) [file pone.0046156.s009.doc]

**Table S1. Correlation between the UIP and out-degree of the nodes by removing each filter from the filtering process in network construction.** None implies that all filters are applied and none is removed from the filtering process.

|  | Age filter | | Risk group filter | | Treatment filter | | None | |
| --- | --- | --- | --- | --- | --- | --- | --- | --- |
|  | cor | p-value | cor | p-value | cor | p-value | cor | p-value |
| MSM | 0.87 | <2.2e-16 | 0.91 | <2.2e-16 | 0.34 | 3.05e-06 | 0.90 | <2.2e-16 |
| Heterosexual | 0.94 | <2.2e-16 | 0.90 | <2.2e-16 | 0.31 | 3.76e-06 | 0.74 | <2.2e-16 |
| IDU | 0.80 | <2.2e-16 | 0.86 | <2.2e-16 | 0.61 | <2.2e-16 | 0.86 | <2.2e-16 |
| All risk groups | 0.90 | <2.2e-16 | 0.87 | <2.2e-16 | 0.55 | <2.2e-16 | 0.83 | <2.2e-16 |
