# Supplementary material for: Combining Epidemiological and Genetic Networks Signifies the Importance of Early Treatment in HIV-1 Transmission
Source: PLoS One. 2012 Sep 28;7(9):e46156. doi: 10.1371/journal.pone.0046156 (PMC3460924; doi:10.1371/journal.pone.0046156)
Supplement: Table S2 — Fraction of removed edges from the genetic network using different genetic thresholds. Each threshold value corresponds to a percentile of the overall distance distribution measured through the phylogenetic tree. (DOC) [file pone.0046156.s010.doc]

**Table S2. Fraction of removed edges from the genetic network using different genetic thresholds. Each threshold value corresponds to a percentile of the overall distance distribution measured through the phylogenetic tree.**

| Thresholds (percentile) | Fraction of removed edges | | | |
| --- | --- | --- | --- | --- |
|  | MSM | Heterosexual | IDU | All risk groups |
| 0.022 (1^st^) | 99.8% | 98.7% | 94.3% | 98.2% |
| 0.031 (5^th^) | 98.0% | 93.1% | 79.3% | 91.6% |
| 0.036 (10^th^) | 95.1% | 85.8% | 66.5% | 84.1% |
| 0.040 (15^th^) | 91.1% | 77.5% | 56.3% | 76.5% |
| 0.042 (20^th^) | 87.2% | 70.9% | 49.7% | 70.5% |
| 0.045 (25^th^) | 82.9% | 63.8% | 43.3% | 64.3% |
| 0.047 (30^th^) | 78.3% | 57.4% | 37.9% | 58.5% |
| 0.050 (35^th^) | 70.8% | 48.4% | 30.5% | 50.1% |
