# Supplementary material for: Combining Epidemiological and Genetic Networks Signifies the Importance of Early Treatment in HIV-1 Transmission
Source: PLoS One. 2012 Sep 28;7(9):e46156. doi: 10.1371/journal.pone.0046156 (PMC3460924; doi:10.1371/journal.pone.0046156)
Supplement: Table S3 — Percentage of edges filtered from the network by applying each different filter and all filters. (DOC) [file pone.0046156.s011.doc]

Table S3. Percentage of edges filtered from the network by applying each different filter and all filters.

|  | Age filter | Risk group filter | Treatment filter | All filters |
| --- | --- | --- | --- | --- |
| MSM | 44.5% | 0.0% | 69.2% | 80.4% |
| Heterosexual | 37.5% | 55.1% | 67.7% | 91.2% |
| IDU | 15.7% | 0.0% | 38.2% | 45.7% |
| All risk groups | 34.7% | 75.0% | 61.1% | 91.3% |
