# Supplementary material for: Combining Epidemiological and Genetic Networks Signifies the Importance of Early Treatment in HIV-1 Transmission
Source: PLoS One. 2012 Sep 28;7(9):e46156. doi: 10.1371/journal.pone.0046156 (PMC3460924; doi:10.1371/journal.pone.0046156)
Supplement: Table S4 — Sensitivity analysis on the “maximum age difference” parameter. (DOC) [file pone.0046156.s012.doc]

Table S4. Sensitivity analysis on the “maximum age difference” parameter.

| Maximum age difference (years) | MSM | | Heterosexual | | IDU | | All risk groups | |
| --- | --- | --- | --- | --- | --- | --- | --- | --- |
|  | cor | removed edges | cor | removed edges | cor | removed edges | cor | removed edges |
| 2 | 0.83 | 94.6% | 0.66 | 97.4% | 0.75 | 80.2% | 0.73 | 97.0% |
| 4 | 0.85 | 90.4% | 0.67 | 95.6% | 0.78 | 67.2% | 0.76 | 95.0% |
| 6 | 0.88 | 86.6% | 0.68 | 94.0% | 0.82 | 57.3% | 0.78 | 93.4% |
| 8 | 0.90 | 83.2% | 0.71 | 92.4% | 0.84 | 50.6% | 0.81 | 92.2% |
| 10 | 0.90 | 80.4% | 0.74 | 91.2% | 0.86 | 45.7% | 0.83 | 91.3% |
| 12 | 0.91 | 78.3% | 0.76 | 90.3% | 0.88 | 43.1% | 0.84 | 90.7% |
| 14 | 0.92 | 76.5% | 0.77 | 89.4% | 0.89 | 41.4% | 0.85 | 90.2% |
| 16 | 0.93 | 75.0% | 0.78 | 88.7% | 0.89 | 40.0% | 0.85 | 89.8% |
| 18 | 0.94 | 73.9% | 0.78 | 88.2% | 0.90 | 39.4% | 0.86 | 89.6% |
| 20 | 0.94 | 73.0% | 0.79 | 87.7% | 0.90 | 39.0% | 0.86 | 89.4% |
